# Numerical study of the effects of bronchial structural abnormalities on respiratory flow distribution

**DOI:** 10.1186/s12938-016-0278-7

**Published:** 2016-12-28

**Authors:** Shen Yu, Jizhe Wang, Xiuzhen Sun, Yingxi Liu

**Affiliations:** 1 State Key Laboratory of Structural Analysis for Industrial Equipment, Dalian University of Technology, Dalian, 116023 China; 2 Otorhinolaryngology Department, The Second Hospital of Dalian Medical University, No. 467, Zhongshan Road, Shahekou District, Dalian, 116024 Liaoning China

**Keywords:** Respiratory tract, Trachea, Bronchi, Biomechanical modelling, Numerical simulation

## Abstract

**Background:**

The anatomical configurations of respiratory tract would be directly associated with their ventilatory function. It is necessary to fully understand the association between airway configurations and their functions as well as the interactions between different airway segments. In this study, we developed a respiratory airway model to investigate the effects of bronchial structural abnormalities on flow distribution in the bronchi and upper airway.

**Methods:**

Derived from computed tomography (CT) scanner data, three-dimensional (3D) finite element (FE) models of healthy human respiratory tracts were developed with anatomically realistic configurations, including the nasal cavity, oral cavity, pharynx, larynx, trachea, and partial bronchi. Abnormal bronchial configurations were built to correspond to four common bronchial diseases. Through numerical simulation, airway configurations of normal and abnormal bronchi were obtained, and flow patterns were compared between normal and abnormal respiratory tracts, as well as the effects of lower airway changes on flow distribution in the upper airway.

**Results:**

The simulation results showed that during inspiration, abnormal bronchial structures can cause flow redistribution in each generation of bronchi and have significant effects on flow distribution in the daughter bronchi of abnormal segments, but no effect on flow distribution of the upper airway. During expiration, abnormal bronchus structures had a remarkable influence on flow distribution in the trachea, while there was no significant difference in flow distribution when airflow passed from the vocal cords and entered the laryngeal cavity.

**Conclusions:**

Therefore, abnormal bronchial structures can affect the downstream flow distribution and cause flow redistribution throughout the entire bronchial branches. During expiration, the configurations of the trachea and glottis can diminish the effects of abnormal bronchial structures on flow distribution.

## Background

The respiratory tract is the channel of the human body connected to the external environment for air exchange, and consists of structures including the mouth, nose, pharynx, larynx, trachea, and bronchi. The anatomical configurations of each segment are directly associated with their ventilatory function. The structural abnormalities of certain segments can affect flow distribution of other segments in the airway and even lead to pathological changes [1–3]. It is necessary to fully understand the association between airway configurations and their functions as well as the interactions between different airway segments, and to develop numerical platforms for investigating the structural respiratory lesions and help clinicians understand the lesions from the biomechanical point of view.

In recent years, domestic and international researchers have investigated flow patterns and related functions of respiratory airways, including the upper airway, trachea, and bronchi, using numerical simulations and experimental methods [4–7]. However, most of this research has only considered the effects of mouth-open breathing or adopted Weibel’s Type A symmetrical airway model [8, 9]. Developing a realistic configuration of the upper airway, trachea, and partial bronchi and using it as a whole system for investigating airway flow patterns has seldom been reported. Starting from normal airway structures, this study developed realistic airway models from computed tomography (CT) scanner data, consisting of the nasal cavity, oral cavity, pharynx, larynx, trachea, and bronchi extending to the fifth generation. On the basis of normal respiratory structures, the configurations of the bronchial tree were modified to build two common bronchial disease models: cancer and bronchostenosis. Flow distribution in abnormal airways was simulated and compared to that of normal airways, and the effects of bronchial abnormalities on flow distribution of the entire airway were analysed.

## Methods

**Fig. 1:**
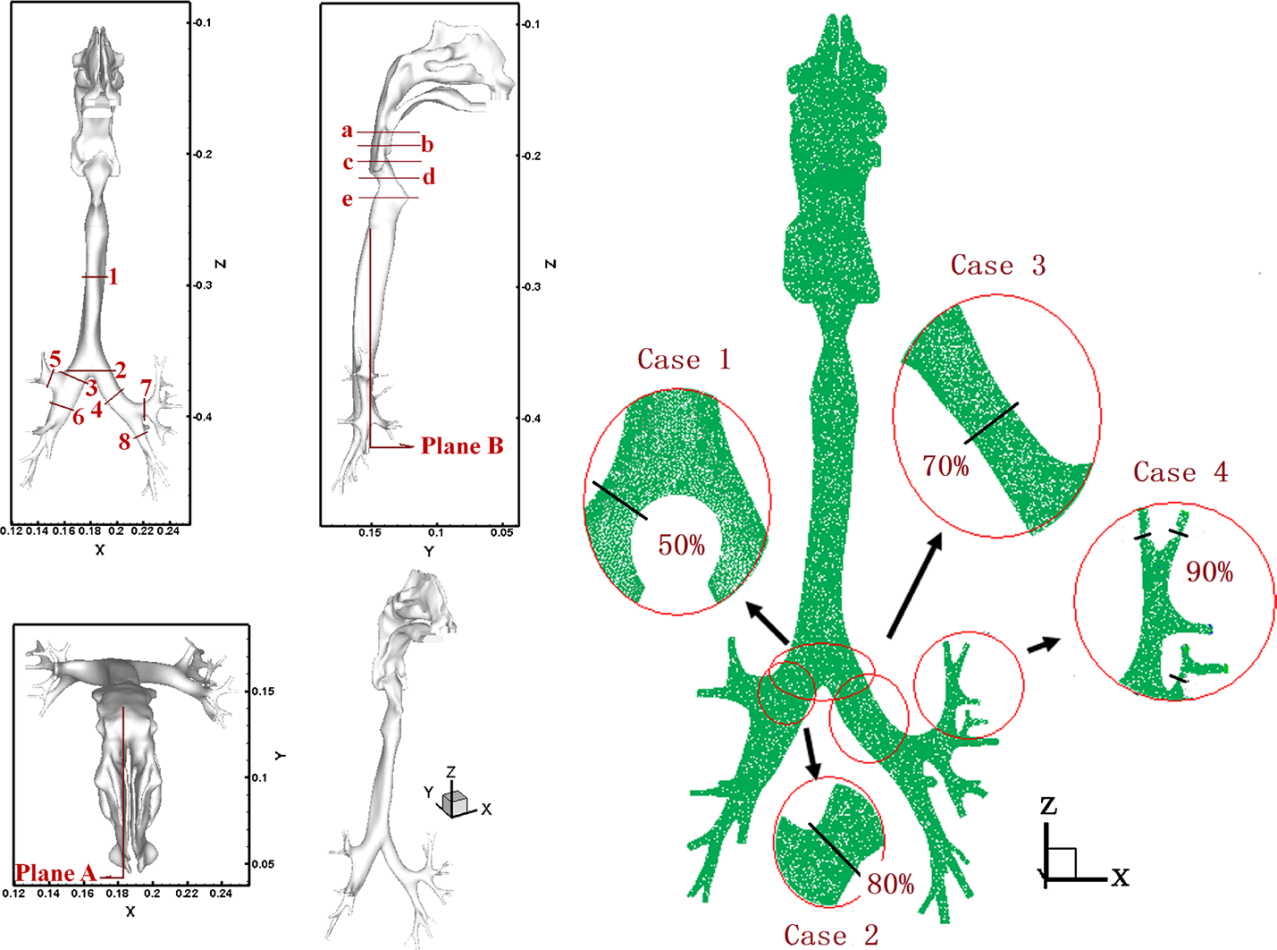
Three dimensional models of normal and abnormal respiratory airway

The CT data were obtained from the Second Affiliated Hospital of Dalian Medical University. The CT scanner version was Siemens Somatom Plus4 Volume Zoom produced by the German Siemens company. The subject was a 35-years-old healthy male without any respiratory medical history last 3 months and no previous history of respiratory trauma and surgery. The subject was informed and agreed to the experiment. This study was approved by Ethics Committee. The volunteer was asked to sit still for 10 min at room temperature. The CT scanning was performed with 1 mm interval and bone tissue window (window width 2000 Hu, window level for the 400 Hu). The images are reconstructed into a 512 × 512 matrix with a resolution of 0.5 × 0.5 mm^2^. Based on CT data from volunteers, a realistic three-dimensional (3D) numerical airway model was developed by the softwares of Mimics, Geomagic and ANSYS-WorkBench, consisting of the nasal cavity, oral cavity, pharynx, larynx, trachea, and bronchi extending up to the fifth generation (Fig. 1). The surface reconstruction for the model was performed by software of Mimics and the model is composed of many triangles. The smoothing and consolidating works for the model was done by software of Geomagic. This model was imported to the software of ANSYS-WorkBench and converted into a 3-D numerical model for the subsequent numerical simulation work. Considering the computing accuracy and time, we eventually adopted a model with a total 3,000,000 elements and 580,000 computational nodes. On the basis of normal airway structures, local airway configurations of the trachea and bronchi were modified to simulate bronchial cancer (case 1), brochostenosis of the right primary bronchus (case 2), brochostenosis of the left bronchus (case 3), and small airway obstruction (case 4). The comparisons between details of the local region and original normal airway configurations are shown in Fig. 1b. The percentages in the figure represented the ratio of cross-sectional area of the narrowest region in the abnormal airway to that in the original normal airway.

The governing equations were Navie-stocks equations. The k-epsilon solver models is used in the simulation. The scheme of SIMPLE is adopted for the pressure–velocity coupling. Boundary conditions were chosen as follows: the nostrils were set as a velocity inlet boundary and a sinusoidal velocity boundary condition was imposed with a 3-s cycle. The tidal volume of respiration was set at 600 ml. After computing the resistances of both nasal cavities, they were essentially equal; therefore, both nostrils were considered as having the same flow rate. A relative pressure condition was placed at the bronchus end (*P* = 0) that was elongated appropriately to limit the assumption of equivalent pressure and the effects on the downstream airway [10]. Complete respiratory tract walls were treated as rigid with no-slip; i.e., *V* = 0 m/s.

## Results

### Velocity profiles of airflow in the normal respiratory track

**Fig. 2:**
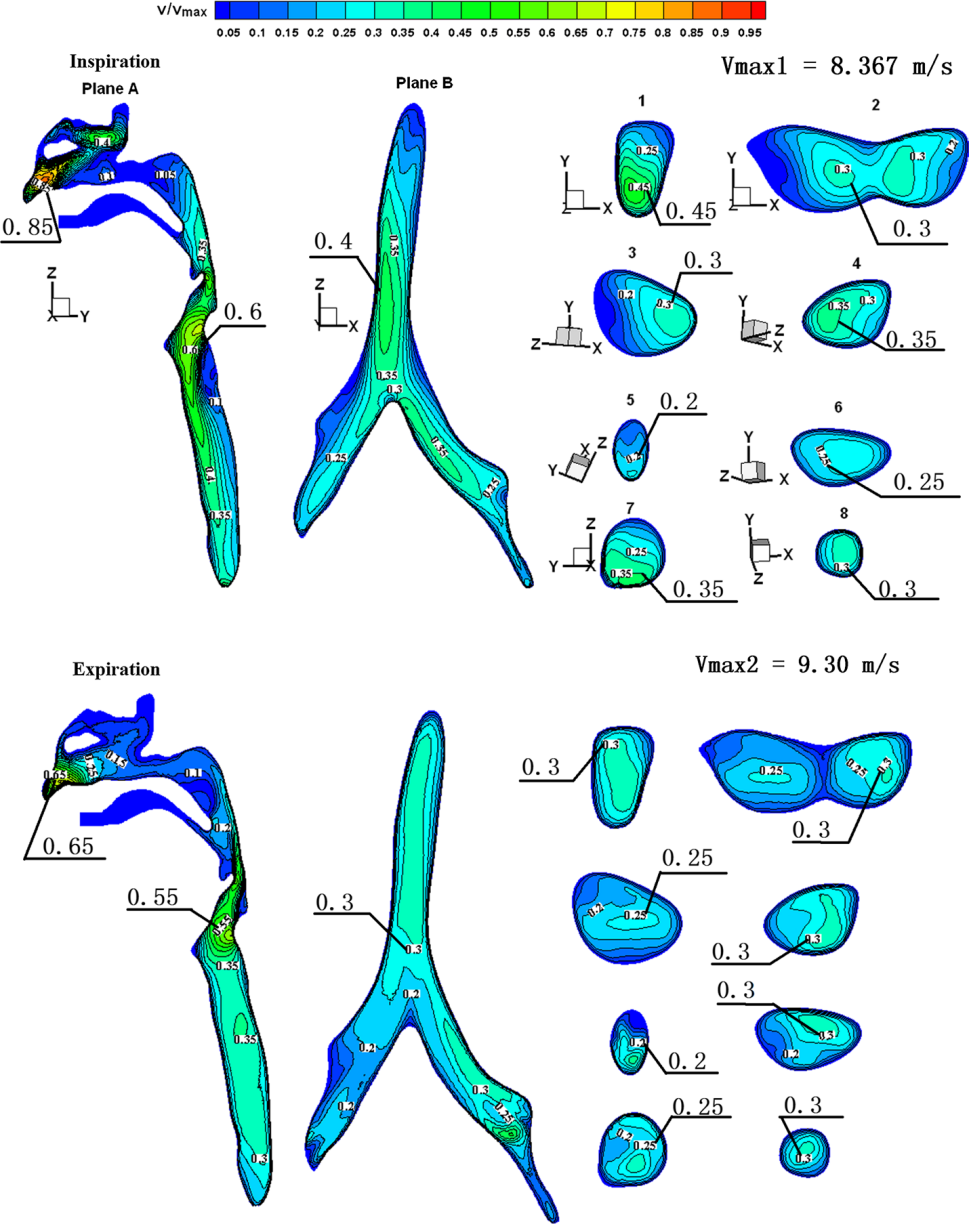
Contour plots of a dimensionless velocity profile of respiratory airway during peak inspiratory and expiratory flows

The airflows of normal airways and the four abnormal airways were calculated within one respiratory cycle. Results at the peak inspiratory and expiratory flows were chosen for comparison. In order to achieve a convenient comparison, results were expressed as dimensionless values. Velocity was non-dimensionalised based on *v*_max_ at the peak inspiratory and expiratory flows. Figure 2 shows the velocity profile of normal airways at the chosen time. The simulation results demonstrated that due to cross-sectional areas of the lumen nasi and glottis being relatively narrow, a high velocity zone was generated in these two regions during respiration. In this model, the trachea had a tubal shape with a narrow opening and a wide end. Therefore, when air passed through the glottis and followed the direction of air duct, a high velocity zone was formed close to the anterior tracheal wall where it was relatively narrow (Plane 1). Plane 2 represents the cross-section of the first bifurcation of the trachea, where airflow was divided into two flows entering the right and left bronchi, respectively, and a high velocity zone was also divided into two from the middle region. As shown in Planes 3 and 4, the high velocity zone, after dividing in two from the middle region, entered the primary bronchus and the high flow rate remained near the inner region of the primary bronchus. Planes 5 to 8 are cross-sections of the secondary bronchus. Considering the spatial position, the secondary bronchus and primary bronchus fell on the same axis, represented by Planes 6 and 8. Therefore, airflow was fully developed when passing Plane 6 and 8, and the flow distribution changed and was similar to secondary distribution with a centred high-velocity zone and a peripheral low velocity zone. An angle formed between the secondary bronchus and primary bronchus, represented by Planes 5 and 7. When airflow passed Planes 5 and 7, a high velocity zone formed near the inner bronchus wall.

### Velocity profiles of airflow in the abnormal respiratory track

**Fig. 3:**
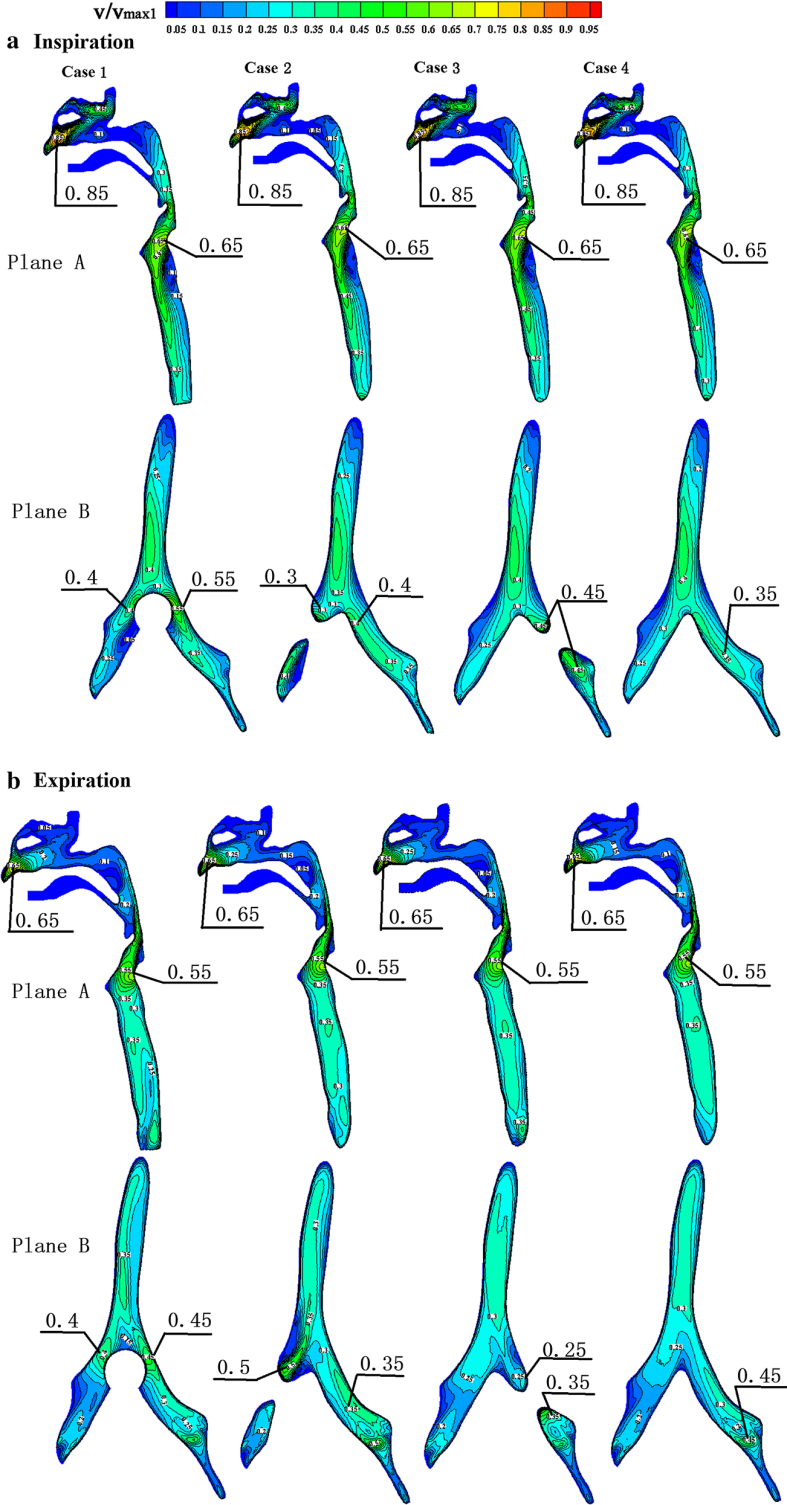
Contour plots of dimensionless airway velocity profiles of four abnormal cases at peak **a** inspiratory and **b** expiratory flows

**Fig. 4:**
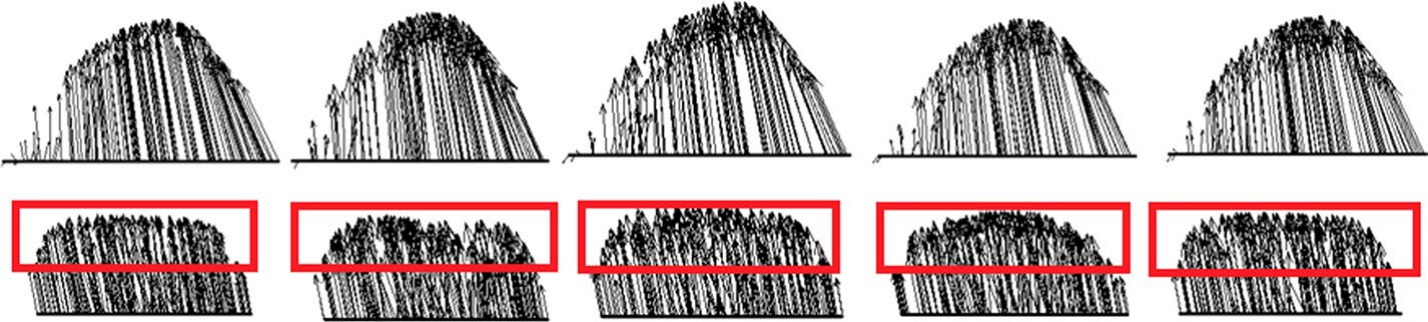
Vector diagrams of velocities in Plane 1 and Plane 2 during expiration

**Fig. 5:**
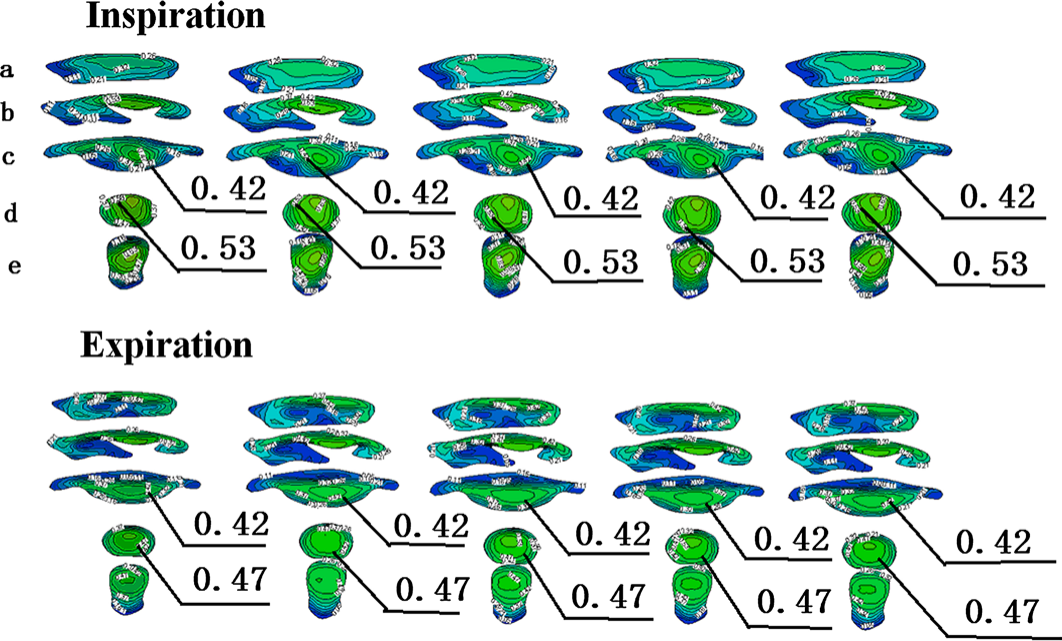
Contour plots of flow velocity profiles in the pharyngeal cavity and laryngeal cavity during inspiration and expiration (Planes *a* to *e*)

Figure 3 shows the velocity profiles in four cases with abnormal airways during peak inspiratory and expiratory flows. During inspiration, there were generally no differences between the flow distribution in the upper airway and trachea compared to normal airways. On the other hand, for the flow velocity profiles of the bronchi, there was a significant difference between the normal bronchi and those of cases 1, 2, and 3, with a relatively smaller difference in case 4. Velocity increased remarkably when the airflow passed the narrow region of the bronchi. During expiration, there was a significant difference in flow distribution of the bronchi between normal airways and those of cases 1, 2, and 3, with a relatively smaller difference in case 4. In all five models, the difference in velocity profiles was comparatively more significant at the moment when airflow entered the trachea from the bronchi. After airflow moved a certain distance in the trachea, velocity profiles were gradually stable and the differences were reduced (Fig. 4). The difference in velocity profiles was further decreased near the glottis (Fig. 4). As shown in Fig. 5, when airflow entered the upper airway through the glottis, the airway flow distributions in the five models were similar, and the degree of similarity in velocity profiles of case 4 was higher than those of case 1, 2, and 3, indicating that the configurations of the trachea and glottis remarkably diminished the effects of bronchial structural abnormalities on velocity profile during expiration.

### Velocity profiles of airflow in the bronchus

According to Table 1, the airflows in both primary bronchi of normal airways were not completely identical. In case 1, there were no significant changes in the flow distribution of both branches. In case 2, the resistance of the right branches increased while airflow decreased, and the airflow of the left branches was consequently increased. In case 3, the resistance of the left branches increased while the airflow decreased, and the airflow of the right branches consequently increased. In case 4, the airflow in the left primary bronchus decreased, while airflow increased in the right primary bronchus.

**Table 1:** Flow distributions in the left and right primary bronchi under five conditions

Flow (ml/s)	Normal		Case 1		Case 2		Case 3		Case 4	
	IP	EP	IP	EP	IP	EP	IP	EP	IP	EP
Left bronchus	311	297	307	290	334	347	290	262	301	289
Right bronchus	289	303	293	310	266	253	310	338	299	311
Total flow	600									

*IP* inspiration, *EP* expiration

**Fig. 6:**
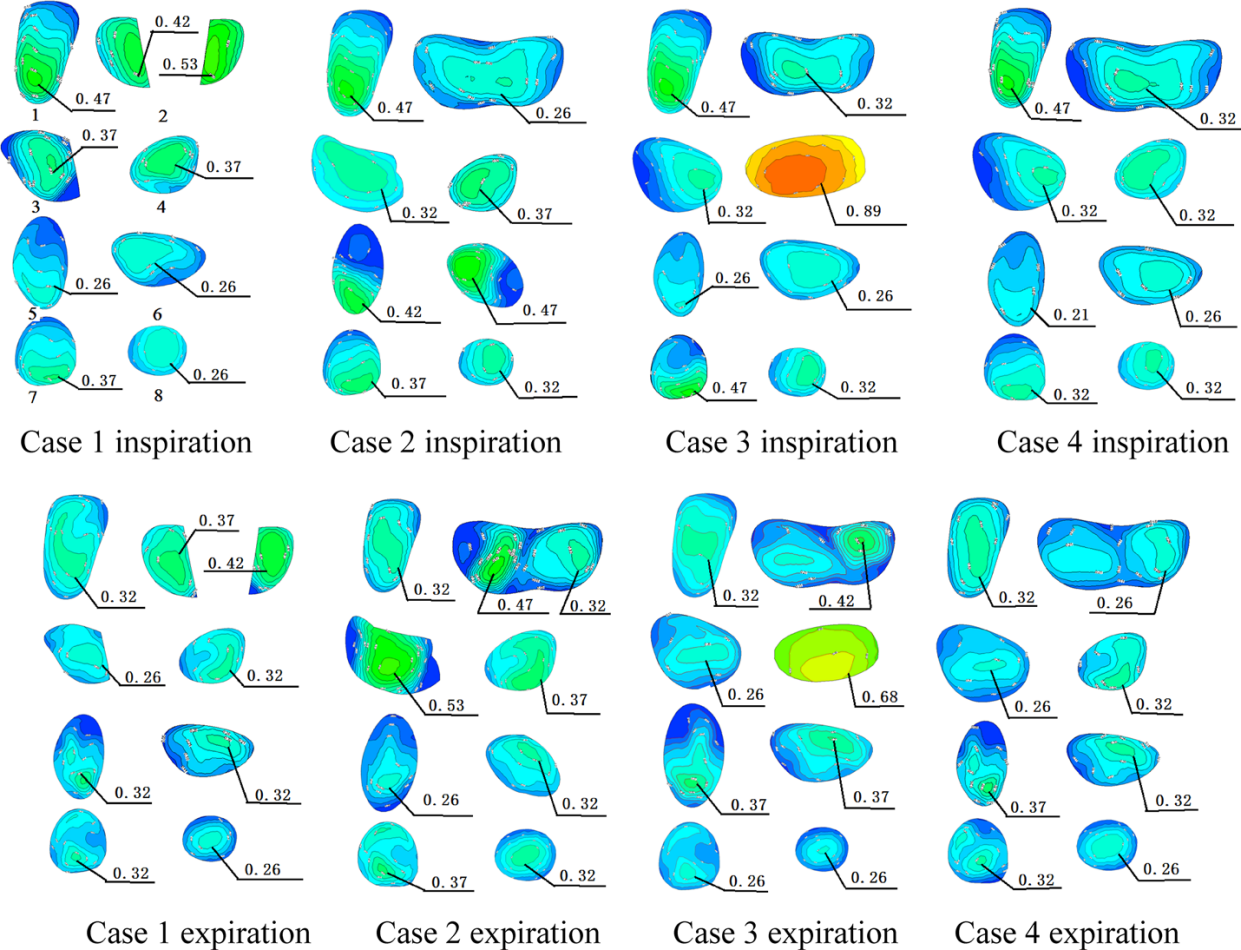
Contour plots of dimensionless bronchial velocity profiles of four abnormal cases during peak inspiratory and expiratory flows

Figure 6 shows the velocity profiles with peak inspiratory and expiratory flows in the trachea and bronchi of the four abnormal case models. Compared to normal airways (Fig. 2), in the trachea of case 1, there were no significant differences in the velocity profiles of Plane 1 during inspiration. In Plane 2, due to the tumour that occupied the airway, a decrease in the cross-sectional area of airway led to an increase in flow rate, which indirectly caused an increase in the maximum flow rate of Planes 3 and 4. During expiration, the cross-sectional area of Plane 2 decreased, causing an increase in flow rate. Due to changes in the configuration of Plane 2, the velocity profiles of the downstream Plane 1 and nearby Planes 3 and 4 differed.

Cases 2, 3, and 4 all had unilateral structural abnormalities of the bronchi. Compared to normal airways (Fig. 2), during inspiration, the total airflow entering the right side decreased in the airway of case 2 due to narrowing of the right bronchus, leading to a decrease in maximum flow rate on the right side of Plane 2. In Plane 3, due to the reduced cross-sectional area, the flow rate increased, indirectly increasing the maximum flow rate in Plane 6. Planes 4, 7, and 8 were from the left bronchus. The maximum flow rate in these planes was slightly increased due to increased airflow; however, there were no significant changes in overall velocity profiles. A remarkable vortex structure formed in Plane 5. During expiration, the increased airflows in Planes 4, 7, and 8 led to a higher flow rate, but there were no significant changes in velocity profiles. In Plane 3, the flow rate increased because of the airway narrowing. Plane 2 is located nearby the downstream component of Plane 3, and therefore the flow rate of the right braches in Plane 2 was significantly higher. Plane 1 is downstream of Plane 2, and therefore its velocity profile was different from that of normal airways. In the bronchi of cases 3 and 4, the changing patterns in flow distribution caused by structural changes were similar to that of case 2, and all these changes can result in alterations in flow distribution near or downstream of the structurally altered region. In case 4, the bronchial structural abnormalities were located in the fifth generation bronchus. As seen in Fig. 6, its influence on the overall bronchial flow distribution was smaller than that caused by structural changes of primary bronchi.

### Pressure distribution of airflow in the respiratory track

**Fig. 7:**
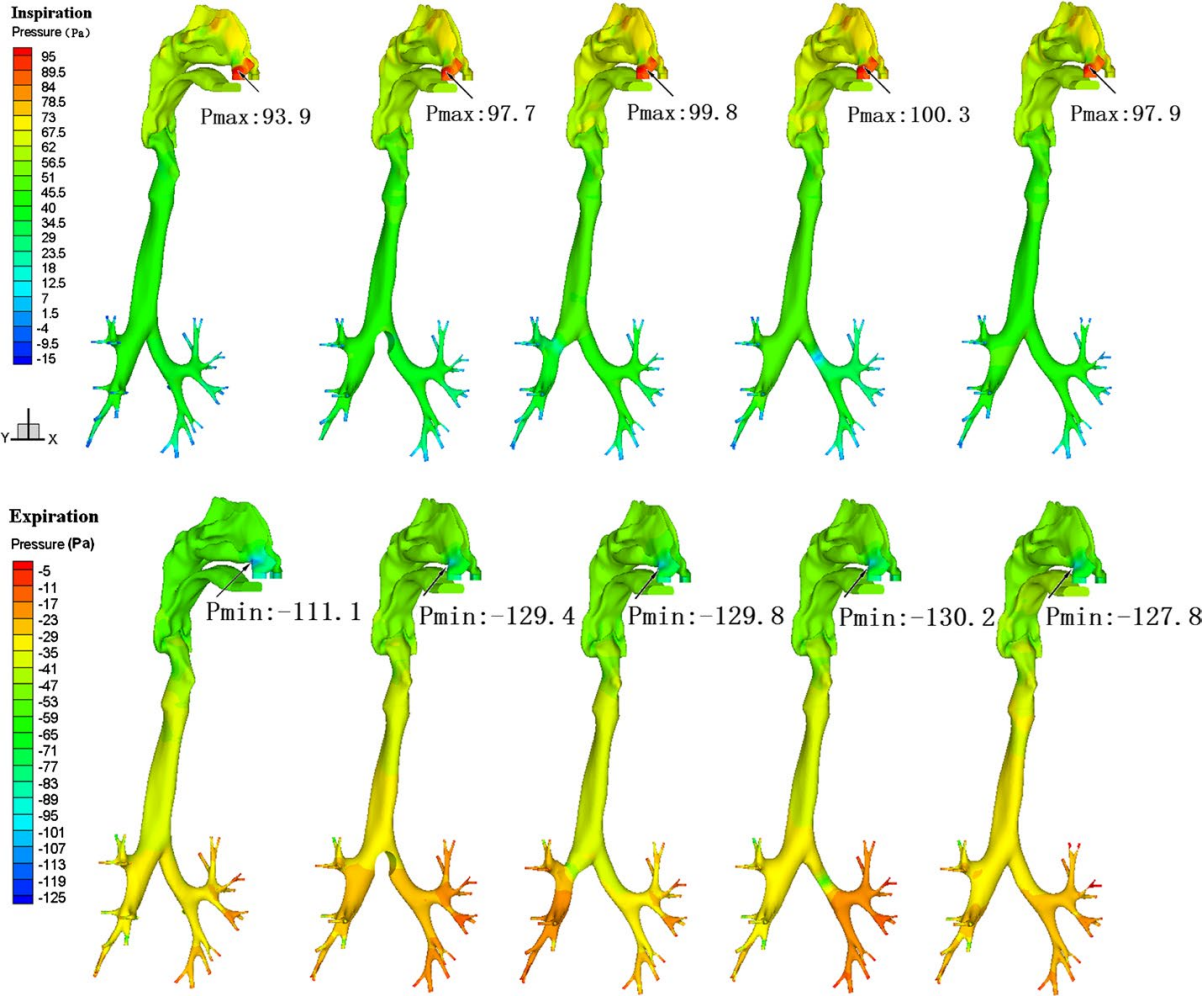
Contour plots of airway pressure distribution profiles during peak inspiratory and expiratory flows

Figure 7 shows the pressure distribution in the airways of five models during peak inspiratory and expiratory flows. From these data, we can conclude that pressure distribution patterns were essentially the same in the five models during inspiration. The total pressure difference in the respiratory tract was approximately 100 pa, with the smallest pressure difference in the normal airway. The most rapid pressure change occurred in the lumen nasi, glottis and the region near the exit of the bronchial tree. The pressure change was very small from the pharyngeal cavity extending to the trachea. There were significant pressure gradients in the regions of the bronchial structural abnormalities in cases 2, 3, and 4. During inspiration, the total pressure difference in the normal respiratory tract was about 110 pa, while the airway pressure differences in the four abnormal cases were about 130 pa, and remarkable pressure gradients appeared in the lumen nasi, glottis, and terminal bronchi. Compared to normal airways, in the airways of the four abnormal cases, the pressure distribution was significantly different in the trachea and bronchi due to flow redistribution in both bronchi.

## Discussion

### Analysis of the effect of respiratory tract structure on its function

From the flow distributions of normal airways and the airways of four cases with structural abnormalities, it can be concluded that during inspiration, abnormal bronchi configurations didn’t affect the velocity profiles in the trachea or the upstream segments of the upper airway. As the flow-restricted segment of the nasal cavity, the lumen nasi has a narrow air duct and thus the maximum flow rate and a large pressure gradient occurred in this region. The glottis is a regional narrow segment of the airway, where flow accelerates down to the trachea, which is consistent with Lin et al’s findings [9]. The orientation of the glottis on airflow directly affected the flow distribution of the trachea. Therefore, although the cross section of the trachea showed a tubal shape with a narrow opening and a wide end, the high flow rate zone in the upper segment of the trachea remained close to the anterior wall of the bronchi. When flow entered two bronchial branches from the trachea, the high flow rate zone was also divided into two and occurred near the inner bronchial wall. This phenomenon was also reported in studies by Adler [11] and GroBe [12]. The maximum flow rate was slightly higher in the left bronchus than in the right bronchus. According to Sebastian et al., this was due to the different angles formed between the two bronchi and trachea, and in fact it was also associated with the resistance of both lungs as well as the flow distributions of both branches. Based on the flow distribution of bronchi on both sides, the airway resistance in both branches were similar and slightly greater in the right airway than in the left airway, which is consistent with Katrin et al’s study results [12]. In secondary bronchi, the lower lobe of the bronchus was the extension of the primary bronchus while the upper lobe of the bronchus formed a 90° angle with the primary bronchus. Therefore, the main flow entered the lower bronchus lobe, which was also observed in a study by Soodt [13]. When the configurations of the trachea and bronchus changed due to disease processes, the resistances of both bronchi were altered correspondingly, and therefore airflow was redistributed in both bronchi. In case 1, the tumour at the bifurcation of the trachea and the primary bronchi reduced the cross-sectional area of both primary bronchi (Plane 2) by 50%. Because the cross-sectional areas of both branches were decreased to the same extent, there were no significant changes in flow distribution in both branches. However, due to the decreased cross-sectional area, the flow rate of the cross section increased. The transverse planes of the primary bronchus (Plane 3 and 4) were close to Plane 2. Therefore, although the cross-sectional area remained the same, the maximum flow rate in this cross section increased. This local change had less effect on the distal regions (such as Plane 8). In case 2, the decreased cross-sectional area of the right primary bronchus led to an increase in airway resistance in the right branches. While the entire respiratory flow remained the same, the flow entering the right airway was reduced when more flow entered the left airway. Due to the decreased cross-sectional area of the right airway, the flow rate increased in both the left and right bronchi (Plane 3 and 4). In case 3, the pattern changes of flow distribution in the bronchus were similar to case 2. In a study by Kim [14], it was shown that narrowing of the right bronchus led to flow redistribution as well as an increase in the maximum flow rate in both bronchi. Case 4 had a bronchostenosis of the fifth generation of the right bronchus (90% of original size). The narrowing of this bronchus caused an increase in total resistance in the right bronchus; thus, airflow decreased in the right branches and the airflow increased in the left branches. Because the narrowing only occurred in one branch of the fifth generation bronchi, there was no significant influence on total airway resistance of the right branches, and therefore fewer changes appeared in both the left and right flow distributions, as well as in the flow rate of both airways. During expiration, flow moved down from the primary bronchus to the trachea and entered the upper airway. The bronchial flow was upstream of the entire airway: the flow distribution of the bronchi can directly affect that of the trachea and further affect the upper airway. The flow from two primary bronchi converge into the trachea and form an M-shaped flow pattern with double peaks in the trachea, which was also observed in the study by Sebastian et al. [13]. After the flow moved a certain distance in the trachea and nearly reached a steady state, the flow pattern changed into a single peak [15]. In the four abnormal cases, structural changes in the local bronchus led to changes in the velocity profile at the bifurcation of the trachea and primary bronchus (Plane 2). The positions of the changes in cases 1–3 were located near Plane 2, and therefore had greater influence on the velocity profile of Plane 2. On the other hand, in case 4, the structural change occurred in the fifth generation bronchus, and therefore the velocity profile of Plane 2 was less affected and was similar to that of the normal case. When flow entered the trachea through the bronchus, the flow velocity profile in the trachea was affected by the flow upstream of Plane 2. Hence, there were significant differences in the velocity profile of the trachea between case 1–3 and the normal case, while the flow velocity profile of the trachea of case 4 was similar to the normal case. The trachea has a length of 10–12 cm, and when flow was moving through the trachea, the velocity profile of the transverse planes will gradually reach a steady state. When flow was passing from Plane 2 to Plane 1, the difference in the flow velocity profile of Plane 1 between cases 1–3 and the normal case decreased. When the flow arrived at the region inferior to the glottis, the difference in the flow velocity profiles of transverse planes was further reduced. The air duct at the glottis is relatively narrow, which can accelerate the flow that passes by. From the glottis to the larynx, the patterns of the flow velocity profiles were very similar and the influence on downstream flow distribution of the nasal cavity was also very small. Therefore, it can be concluded that one of the functions of the trachea and glottis structures is to eliminate the influence from the changes of the altered flow distribution caused by structural abnormalities of the bronchus, on the flow distribution in the upper airway. Ma [16] found that in each generation of bronchial branches, the flow distribution tended to reach a stabilised distribution within a short distance, which might explain the above phenomenon.

From the pressure distributions in the normal airway and the abnormal airways of the four cases, it was apparent that the pressure difference in the nasal cavity was approximately 50% of the entire respiratory tract during expiration and the largest pressure gradient was found in the lumen nasi. A relatively remarkable pressure gradient also occurred in the glottis. The overall pressure distributions in all cases were essentially similar, and the larger pressure gradients were only found in cases 2–4 at the region of the narrowed bronchi. There were no significant differences in the pressure difference between normal airways and the abnormal airways of the four cases. In case 1, the tumour occupied 50% of the cross-section of the airway at the bifurcation of the trachea and primary bronchus. However, there were no significant pressure gradients, indicating that the air duct was relatively wide at the bifurcation. Even though the cross-sectional area had changed to certain degree, the ventilatory function remained relatively unaffected. For cases 2 and 3 with unilateral narrowing of a local primary bronchus, because the left and right primary bronchi are parallel airways, when the narrowing occurred in the unilateral local region to a limited extent (70% in left, 80% in right), the total resistance was not greatly affected and the total pressure drop was only slightly higher than that of the normal case. Case 4 had a bronchostenosis of the fifth generation bronchus. The fifth generation bronchus has a limited influence on the overall airway resistance, and therefore pressure differences in case 4 were only slightly higher than that of the normal case, while it was lower than that of cases 2 and 3. During expiration, the pressure distribution was similar to the pressure gradient pattern during inspiration, and the pressure gradient was more significant at the local stenosis in the airway, such as the lumen nasi, glottis, and the bronchial stenosis of the abnormal airway.

It can be concluded from the above that there were some common patterns in airway flow distribution. The narrowness of unilateral bronchi led to an increase in airway resistance in the same branches and decreased airflow. Meanwhile, due to the decreased area of the narrowed transverse planes, the flow rate in the planes was increased, as did the pressure gradients in the adjacent area. Meanwhile, the airflow increased in the airway of the other side, and therefore the total flow velocity increased. The influence of the narrowed unilateral airway on the flow distribution of both airways was related to the location of the stenosis. The bronchostenosis of a higher bronchial generation would have greater effects on the flow distribution ratio for both sides. During expiration, local bronchostenosis can affect the flow velocity profile in the trachea, and the effects can be largely diminished by the narrow and long configurations of the trachea and glottis. After airflow entered the laryngeal cavity, the velocity profiles of abnormal cases were almost the same as in the normal case.

## Limitations

Some limitations of this study have to be discussed. First, in this paper, only one numerical model was made. Due to the difference in the structure of human body, additional cases will be investigated in the future, which will make the conclusions more general. Second, the model contains only a small portion of the bronchus, due to the limitation in the resolution of the CT used in current study. Developing bronchial models from higher resolution CT images will better reflect the mutual influence between the various parts of the respiratory tract. Finally, in the numerical simulation of airflow in the respiratory tract, the turbulence model was selected. In the nasal and pharyngeal cavity, the airflow is transitional flow or turbulence flow, and in the bronchi the airflow should be laminar flow, which is determined by the function of different parts of the respiratory tract. We are continuing our efforts to solve the above problems to get more reliable results.
